# Genetic variability of *Aedes aegypti* (Diptera: *Culicidae*) in El Salvador and Honduras: presence of a widespread haplotype and implications for mosquito control

**DOI:** 10.1186/s13071-024-06312-7

**Published:** 2024-05-16

**Authors:** A. L. Joyce, Miguel Moreno, Leonel Palomo, Raul O’Connor, Denis Escobar

**Affiliations:** 1 Public Health, University of California, 5200 North Lake Road, Merced, CA 95343 USA; 2 Departmento de Biología, Final de Av. Mártires y Héroes del 30 Julio, University of El Salvador, San Salvador, El Salvador; 3 Unidad de vigilancia de la Salud, Secretaría de Salud de Honduras, Tegucigalpa, 11101 Honduras; 4 Microbiology Research Institute, Universidad Nacional Autónoma de Honduras, Tegucigalpa, 11101 Honduras

**Keywords:** *Aedes aegypti*, Haplotypes, Mitochondrial DNA, Central America

## Abstract

**Background:**

This study examined population genetics of *Aedes aegypti* in El Salvador and Honduras, two adjacent countries in Central America. *Aedes aegypti* is associated with yellow fever, dengue, chikungunya, and Zika. Each year, thousands of cases of dengue are typically reported in El Salvador and Honduras.

**Methods:**

In El Salvador, collections were obtained from five Departments. In Honduras, samples were obtained from six municipalities in four Departments. Mitochondrial DNA cytochrome oxidase I (COI) was sequenced, and consensus sequences were combined with available sequences from El Salvador to determine haplotype number, haplotype diversity, nucleotide diversity, and Tajima’s D. A haplotype network was produced to examine the relationship between genotypes.

**Results:**

In El Salvador, there were 17 haplotypes, while in Honduras there were 4 haplotypes. In both El Salvador and Honduras, Haplotype 1 is most abundant and widespread. In El Salvador, haplotype H2 was also widespread in 10 of 11 sampled municipalities, but it was not present in Honduras. The capital of El Salvador (San Salvador) and the eastern region of ES had the highest haplotype diversity of regions sampled.

**Conclusions:**

Haplotype 1 and H2 each belong to different phylogenetic lineages of *Ae. aegypti*. The most geographically widespread haplotype (H1) may have been present the longest and could be a remnant from previous eradication programs. These data may contribute to future control programs for *Ae. aegypti* in the two countries.

**Graphical Abstract:**

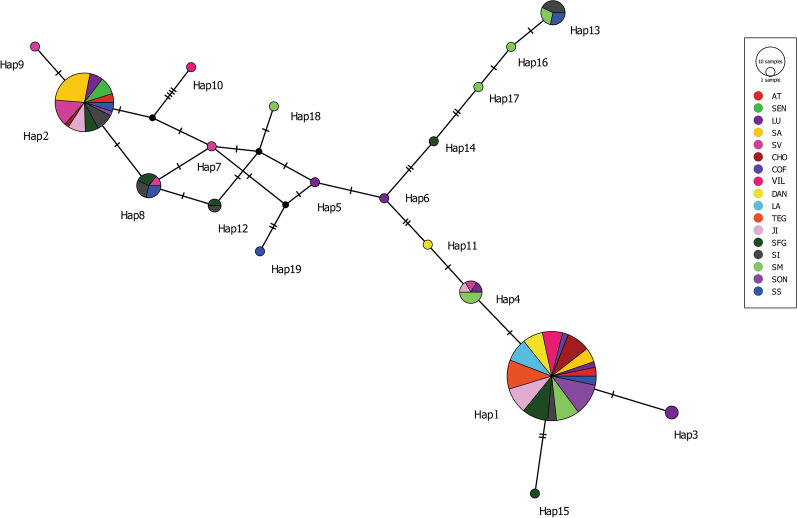

**Supplementary Information:**

The online version contains supplementary material available at 10.1186/s13071-024-06312-7.

## Background

*Aedes aegypti* is one of the primary vectors of dengue, Zika, chikungunya, and yellow fever viruses. The mosquito originates from Africa, has been transported globally, and is now established in many tropical, subtropical, and even temperature regions [1, 2]. In addition to vectoring infectious disease, this mosquito is associated with human habitation. Areas with artificial water storage around homes and buildings provide ideal breeding sites, making this species a pest globally [1].

*Aedes aegypti* populations from Africa group into two mitochondrial lineages, and globally the two lineages are widely distributed with genetically distinct populations [3]. Population genetic studies have found that insects including mosquitoes have divergent populations or strains, which vary in their vector competence [4–6]. Populations of *Ae. aegypti* can survive a range of environmental conditions [7]. Determining the population genetics of a species, and the distribution of biotypes and strains and their biological attributes, can contribute to management of vectors and disease prevention. Currently, novel control approaches for *Ae. aegypti* such as sterile insect technique (SIT) and *Wolbachia*-infected mosquitoes used for incompatible insect technique (IIT) are being developed, implemented and evaluated worldwide [8, 9]. These innovative approaches, along with insecticide control of mosquitoes, may benefit from knowledge of the genetic types or strains of *Ae. aegypti* present in a region.

While *Ae. aegypti* was previously eradicated in much of the Western Hemisphere in the 1950s–1960s, including in Central America in El Salvador and Honduras, it has since reemerged as a public health threat [10–14]. *Aedes aegypti* was reintroduced and periodic outbreaks of dengue have been occurring since 1980 [11]. The mosquito is currently widespread in El Salvador and Honduras [15, 16]. In Central America, 4 to 5 million people annually are estimated to be infected with dengue [17]. In 2011–2013, over 29,000 cases of dengue were reported in El Salvador [18]. From 2005 to 2014, El Salvador reported 238,232 cases of dengue and Honduras 268,411, without considering the additional public health burden of Zika and chikungunya which emerged in 2015 [19]. *Aedes aegypti*-transmitted infectious disease cases have continued to increase in recent years in both countries as well as in South America [20, 21], and effective vector control interventions for this mosquito in this region are limited yet remain of the utmost importance [15–18].

Population genetics of *Ae. aegypti* have been investigated globally, within the Western Hemisphere, and a few regional studies have been conducted in Central America. In Central America, a study in Panama found that *Ae. aegypti* had high genetic diversity and primarily grouped into two genetically divergent clusters associated with wet and dry climates [22, 23]. In El Salvador, populations of *Ae. aegypti* from six regions were similarly found to consist of two lineages and a high genetic diversity [15]. In contrast, a study in Honduras of *Ae. aegypti* uncovered few haplotypes, in contrast to other studies in the Western Hemisphere [16].

The objective of this study was to further examine the population genetics of *Ae. aegypti* in El Salvador and in Honduras, as they are two adjoining countries in Central America. El Salvador is on Pacific Coast of Central America, while Honduras borders the Caribbean on the east coast. Both countries have major port cities with international commerce and movement of goods and international tourism and migration. New collection sites were obtained from El Salvador and Honduras for examining *Ae. aegypti* genetic diversity. The number of lineages, genetic haplotypes, and nucleotide diversity of *Ae. aegypti* will be compared between samples from the two countries.

## Methods

**Fig. 1﻿:**
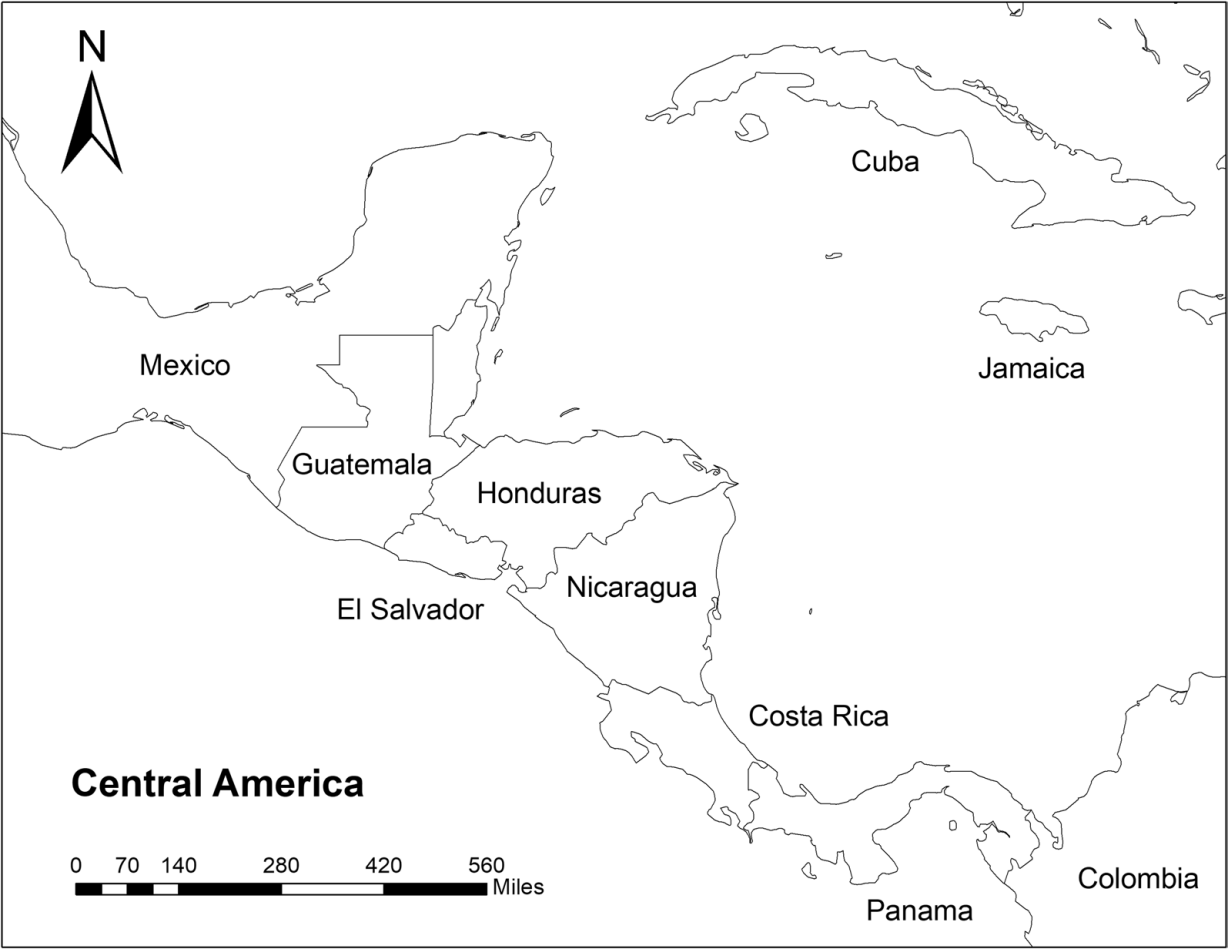
Map of Central America including El Salvador and Honduras adapted from ArcGIS 10.8 shape file for Central America

**Fig. 2:**
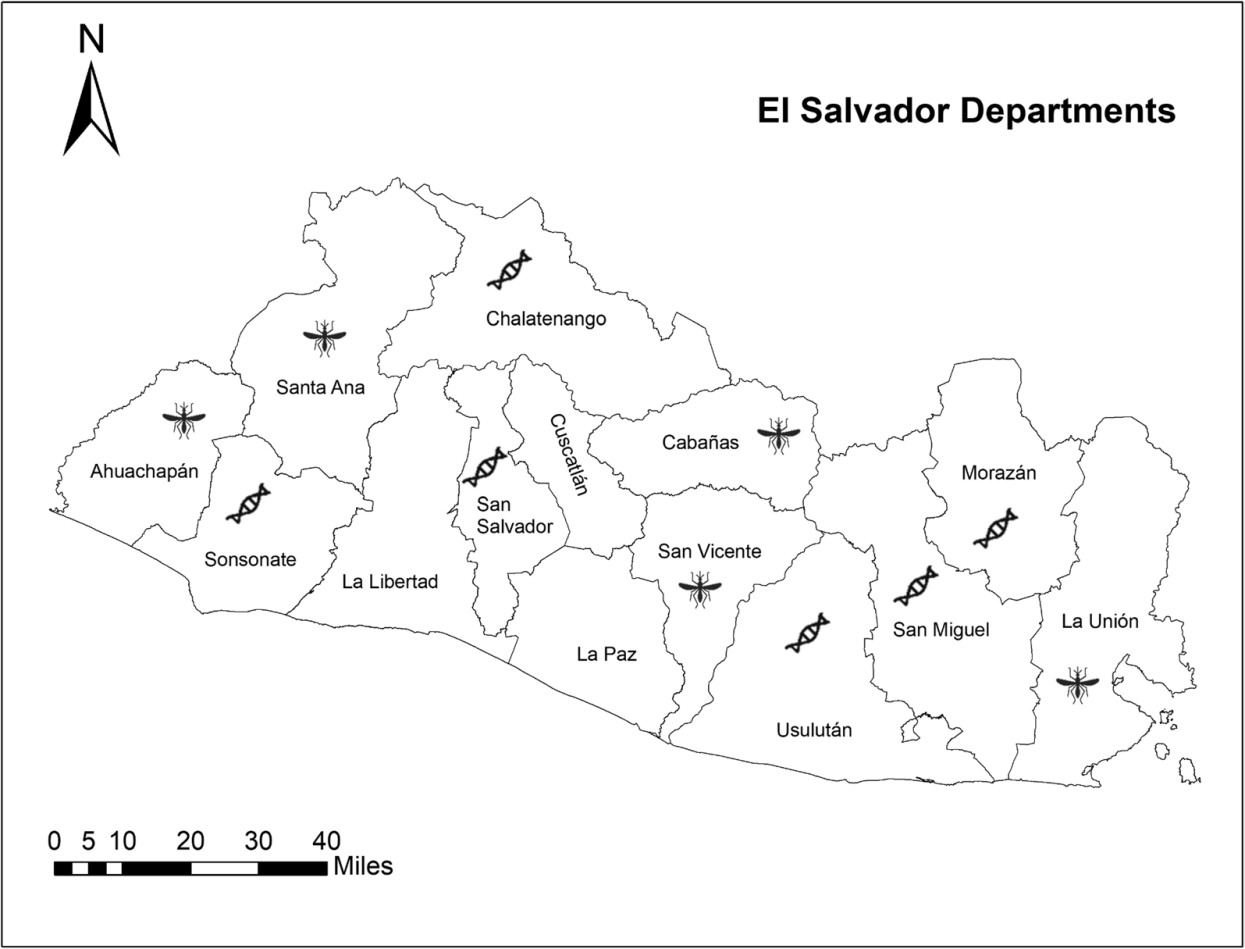
Map of El Salvador with boundaries of each Department. New samples were obtained from five departments indicated by a mosquito icon, and existing sequences were available from an additional six departments and are represented by a DNA sequence. Overall, data used came from 11 of 14 departments throughout the country. Map is modified from ArcGIS 10.8 shape file for El Salvador

**Fig. 3:**
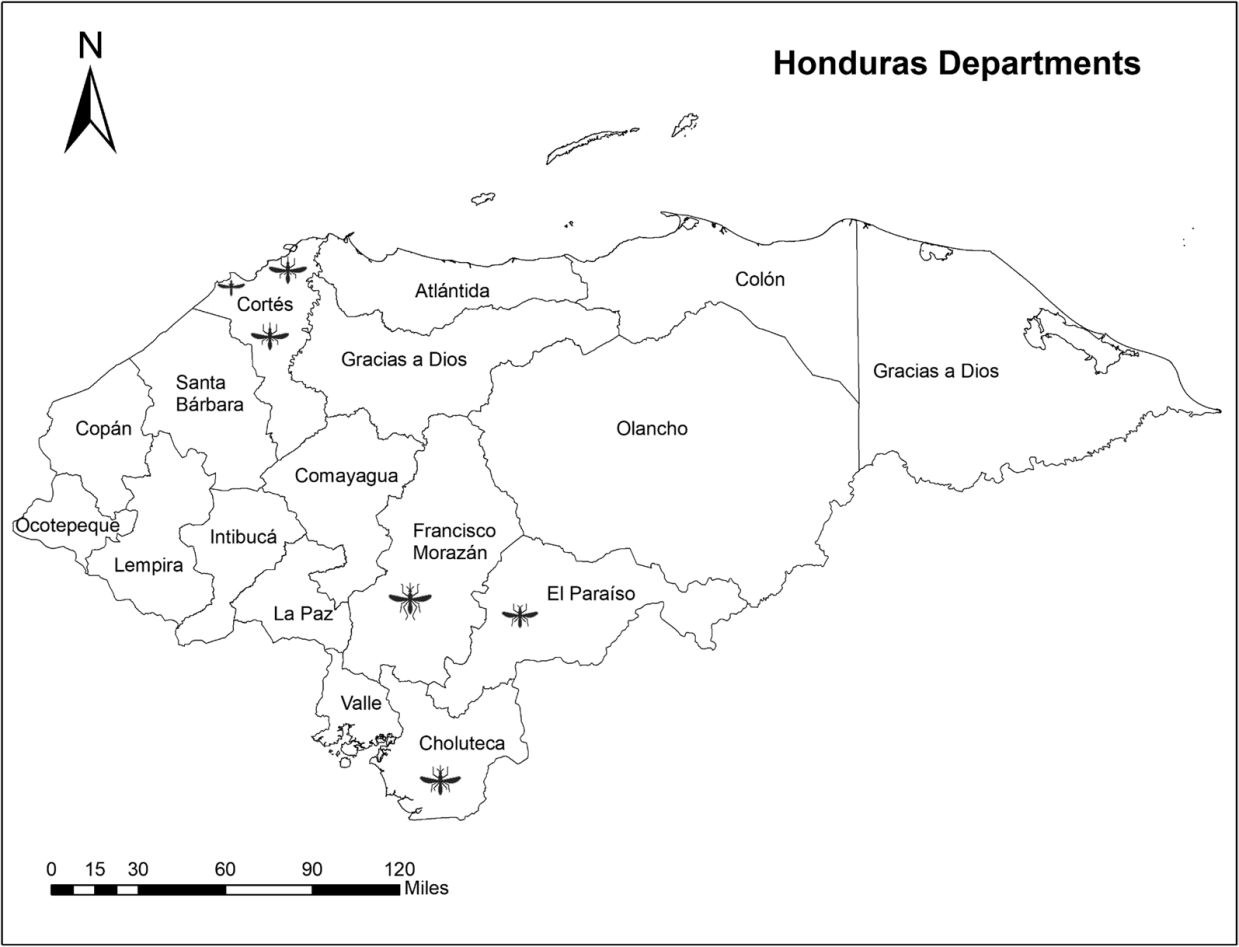
Map of Honduras with boundaries of departments. Samples were included from six municipalities in four departments, Cortés, Francisco Morazán, El Paraiso, and Choluteca, indicated with a mosquito icon (see Table 1). Map is modified from ArcGIS 10.8 shape file for Honduras

**Study Sites** Collection sites were in El Salvador and Honduras, Central America (Figs. 1, 2, 3). Samples were collected in El Salvador from January to March 2018. Larvae were collected in the following five Departments: Santa Ana, Ahuachapan, Cabañas, San Vicente, and La Unión (Table 1, Fig. 2). These municipalities were selected to obtain data on genotypes of *Ae. aegypti* present in additional regions of the country which had not been previously sampled [15]. Santa Ana is a department on the border of Guatemala and the most populated city in the west of the country. Ahuachapan also borders Guatemala, and collections were made at 1240 m above sea level. Samples in the department of Cabañas came from Sensuntepeque in the northeastern part of the country, a city geographically isolated from the rest of the territory. The collections made in San Vicente were concentrated in communities near the Pan American highway and near the Lempa River. Finally, La Unión is an old port city, located on the coast of the Gulf of Fonseca, with hot arid weather conditions (Table 1, Fig. 2).

**Table 1:** Collection sites for *Aedes aegypti* from El Salvador and Honduras

Country, department	Municipality	City/neighborhood	Sample numbers used	Collect date	Latitude	Longitude
El Salvador						
Sonsonate	Sonsonate	El Carmen	SON 4, 5	Aug.18 2014	13°43′40.58″N	−89°44′1.40″E
			SON 6–9		13°43′42.55″N	−89°44′6.11″E
			SON 13		13°43′43.17″N	−89°44′6.94″E
		San Antonio	SON 17		13°43′40.11″N	−89°44′5.17″E
			SON 22		13°43′38.62″N	−89°44′4.89″E
			SON 23, 25, 26		13°43′36.17″N	−89°44′6.39″E
San Salvador	San Salvador	La Fosa	SS1, 3	May 9, May 16, 2014	13°43′14.42″N	−89°11′55.67″E
			SS4, 5		13°43′14.70″N	−89°11′56.97″E
			SS 9		13°43′14.87″N	−89°11′55.42″E
			SS10, 11		13°43′13.69″N	−89°11′56.24″E
			SS16		13°43′14.03″N	−89°11′52.76″E
		San Jacinto	SS 24, 25		13°41′5.44″N	−89°10′46.94″E
Chalatenango	San Ignacio	La Villa	SI 1–4	Aug. 29, 2014	14°20′21.11″N	−89°10′44.58″E
			SI 6, 7		14°20′19.69″N	−89°10′44.58″E
			SI 20		14°20′17.69″N	−89°10′40.87″E
			SI 25–26		14°20′18.52″N	−89°10′39.35″E
			SI 28		14°20′18.24″N	89°10′37.90″O
Usulután	Jiquilisco	Las Flores Siembras	JI 1, 2	June 5 2014	13°19′33.88″N	−88°34′18.50″E
			JI 4		13°19′32.11″N	−88°34′16.48″E
			JI 7–9		13°20′8.87″N	−88°34′21.86″E
			JI 14		13°20′11.69″N	−88°34′22.42″E
			JI 15, 17		13°19′37.89″N	−88°34′12.80″E
San Miguel	San Miguel	Las Americas	SM 1–4	June 5 2014	13°28′41.55″N	−88°10′19.34″E
			SM 6–11		13°28′40.85″N	−88°10′22.68″E
			SM 18–23		13°28′40.76″N	−88°10′20.14″E
Morazán	San Francisco de Gotera	San Francisco de Gotera	SFG 1–3	July 27 2014	13°41′7.87″N	−88° 5′54.10″E
			SFG 4, 5, 9, 10		13°41′7.30″N	−88° 5′53.84″E
			SFG 11–13		13°41′8.19″N	−88° 5′56.27″E
			SFG 16–17		13°40′54.08″N	−88° 5′59.03″E
			SFG 18, 19		13°40′54.51″N	−88° 5′58.76″E
			SFG 20, 22, 23		13°40′54.84″N	−88° 6′0.29″E
			SFG 24–26		13°40′52.35″N	−88° 6′3.14″E
Santa Ana	Santa Ana	Col.Altos del Palmar	SA 1, 3–5, 7–10, 12	Jan 25 2018	13°58′06″N	−89°3′27´W
		Col.El palmar, Pje.Bolivia	SA 22–25, 28		13°58′42″N	−89°34′00″W
		Calle 37	SA 30		13°58′42″N	−89°34′00″W
		Cton.El Ranchador	SA 31		14°00′54″N	−89°36′27″W
San Vicente	San Vicente	Cton.rio Frio	SV 44, 46, 48–50	Feb 2 2018	13°36′12″W	−88°36′12″W
		San Fran. Chanmoco, Crio.valle Nuevo	SV 56, 58		13°36′12″W	−88°39′33″W
		Crio. Junquillal	SV 59, 68, 75		13°38′33″N	−88°43′15″W
Cabañas	Sensuntepeque	Parque Cabañas	SEN 83	Feb 6 2018	13°52′48″N	−88°37′46″W
		Col.Quintero	SEN 100, 104, 106		13°53′06″N	−88°37′50″W
La Unión	La Unión	B.Concepción	LU 125, 132	March 5 2018	13°20′08″N	−87°50′58″W
		Col. Beltran	LU 134, 135		13°20′00″N	−87°50′52″W
	Santa Rosa de Lima	C.altos del Estadio	LU 149, 156		13°38′01″N	−87°53′26″W
		B.Las Delicias	LU 158, 160–162		13°37′53″N	−87°53′03″W
Ahuachapán	C. de Ataco	Col.Estrella	AT 250, 255, 259, 261, 264	March 20 2018	13.87405 N	−89.8544 W
Honduras						
Francisco Morazán	Tegucigalpa	Tegucigalpa	TEG 61–70	May 19, 2019	14°05′02″N	87°12′17″W
Cortés	San Pedro Sula	Los Angeles	LA 81, 83, 84, 86–90	June 2019, May 6, 2019	15°28′44″N	88°09′51″W
	San Pedro Sula	Cofradia	COF 41, 44		15°28′44″N	88°09′51″W
	Villanueva	Villanueva	VIL 1–5, 6, 8, 10		15°19′33″N	87°59′28″W
El Paraiso	Danli	Danli	DAN 11–14, 16, 17, 19, 20	May 20, 2019	14°01′25″N	86°33′22″W
Choluteca	Choluteca	Choluteca	CHO 101–110	May 7, 2019	13°18′03″N	87°10′16″W

In each department, one or two neighborhoods were typically visited to collect larvae, with at least two or three collection sites per neighborhood (Table 1). Sample sites included barrels of standing water and outdoor sinks called pilas. The samples at each site typically consisted of a few larvae of the numerous larvae which were present to reduce the chance that the larvae collected were siblings. A previous study found that the mean number of families represented per oviposition site for *Ae. aegypti* was 4.7 [24]. After collection, larvae were transported to the laboratory for rearing into adults at the University of El Salvador. Adults were identified to species using taxonomic keys [25], and females were used for DNA extraction.

Additional sequences included in analyses came from a previous study in El Salvador [15], which collected samples between May and August 2014 (Table 1). Larvae of *Ae. aegypti* were collected in six departments: Sonsonate, San Salvador, Chalatenango, Usulután, San Miguel, and Morazán (Fig. 2). From the prior study of six departments, 82 sequences from GenBank were included in analyses [Table 3, GenBank accessions, Usulatán (MK028219-32), Morazán (MK028233-52), Chalatenango (MK028253-62), San Miguel (MK028263-78), Sonsonate (MK028279-90), and San Salvador (MK028291-300)].

From Honduras, samples came from six collection sites in four departments. In Cortés, three neighborhoods were included, while the other departments (Francisco Morazán, El Paraíso, and Choluteca) each had one neighborhood sampled (Table 1, Fig. 3). The sites were located along a central transportation corridor in Honduras which runs through several types of ecosystems. Cortés department is a tropical region in proximity to the Caribbean Sea. Tegucigalpa is the capital city at 990 m, which has dry tropical forest landscape, and Choluteca is closer to the Pacific (Fig. 3). Colonies were formed from the initial collections of eggs from ovitraps. Eggs in initial collections were reared to adults and then identified using a taxonomic key [25]. In 2018, eggs were randomly sampled from colonies to obtain a sample of 10 eggs from each population, which were individually extracted to obtain DNA.

## DNA extraction

In El Salvador, individual adult females (*n* = 45) were used for DNA extraction, while from Honduran samples, DNA was extracted from eggs (*n* = 45) (Table 1). DNA extraction was completed with the Qiagen DNEasy^®^ Blood and Tissue Extraction Kit following standard protocols [26] and using an overnight incubation of samples at 65 °C. The quantity of DNA in each sample was measured using the Qubit 2.0 fluorimeter Hs DNA kit (ThermoFisher, Waltham, MA, USA).

## Mitochondrial DNA COI

For each insect, DNA was used to sequence a ~ 650-bp region of mitochondrial DNA cytochrome oxidase 1 (CO1) (known as the ‘bar code’) using a universal forward primer LCO 1490 (5′-GGTCAACAAATCATAAAGATATTGG-3′) and reverse primer HCO2198 (5′-TAAACTTCAGGGTGACCAAAAAATCA-3′) [27, 28]. A polymerase chain reaction (PCR) mix for each sample consisted of the following: 40 µl sterile ultra-pure water, 1 µl Taq polymerase (Clonetech, Mountainview, CA), 5 µl Taq 10 × buffer, 1 µl dNTPs, 1 µl forward primer, and 1 µl reverse primer, and each reaction had 2 µl template DNA added. The PCR program was the following: an initial 1 min warm-up at 95 °C; then 40 cycles of a touchdown program consisting of 92 °C for 30 s, 43–52 °C for 30 s (with a 0.3 °C temperature increase each s), and 72 °C for 60 s; after 40 cycles, a 68 °C final extension for 10 min and then a hold at 4 °C. Samples were cleaned up using the Exo-sap-it (Affymetrix, Inc., Santa Clara, CA) cleanup kit and run on a 3730 Genetic Analyzer.

## Data analyses

Resulting sequences were analyzed using Geneious software v.9.1.6 to produce consensus sequences [29]. Sequences were trimmed, forward and reverse sequences were aligned, and a consensus sequence was produced. Sequences were aligned in Geneious and exported to Mega for further analysis [29–31].

The number of segregating sites (S), number of haplotypes (H), haplotype diversity (Hd), nucleotide diversity (Pi), and Tajima’s D were generated using DnaSP v.6.12.03 [32]. Tajima’s D was calculated to test whether there was a departure from neutrality, such as a population expansion or contraction. A median joining haplotype network tree of *Ae. aegypti* was constructed using PopART [33].

The pairwise *Fst* was calculated in Arlequin v.3.5.2 to estimate population differentiation based on differences in haplotype frequencies [34]. Analysis of molecular variance (AMOVA) was conducted to examine the distribution of genetic variation within and among populations using 1000 permutations with Arlequin v.3.5.2 software, and additional analyses were also run to examine variation within each country [34, 35].

## Results

**Fig. 4:**
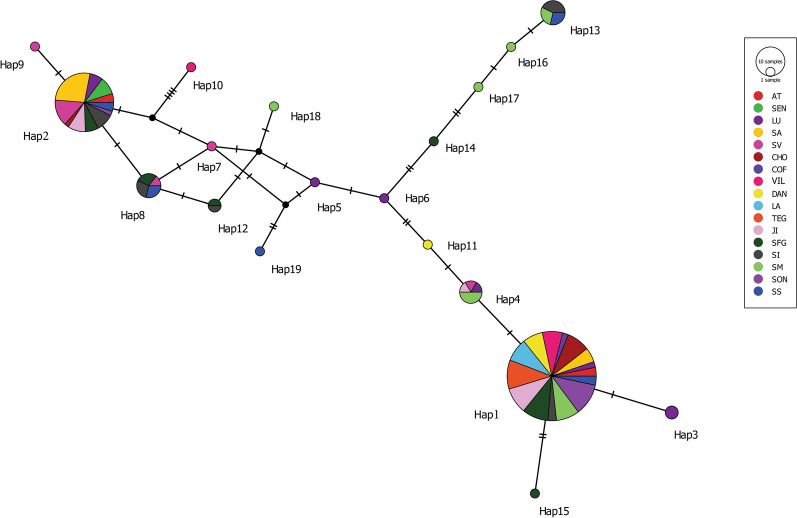
Median joining haplotype network tree of *Aedes aegypti*. The tree was constructed using 19 haplotype CO1 mtDNA sequences from 17 sites in El Salvador and Honduras. The size of each circle is proportional to total number of samples of each haplotype. Black circles represent missing or unsampled steps. The site name abbreviations are described in Table 1

Ninety new sequences were produced, 45 from El Salvador and 45 from Honduras (Tables 1, 2). These sequences were combined with 82 existing sequences from El Salvador for an analysis of 172 sequences from 11 municipalities in El Salvador and 6 municipalities in Honduras (Table 2). A total of 19 different haplotypes were identified in the two countries (Table 3, Fig. 4). When combining all samples from both countries, the higher frequency, broadly geographically distributed haplotypes were H1 (*n* = *94/172*) and H2 (*n* = *40/172*) (Fig. 4). The haplotype network shows the genetic relationships among the haplotypes. Of the 19 haplotypes, 12 (63.2%) were unique to single populations, and 7 haplotypes (36.68%) were shared among populations. Of the 19 haplotypes in the study, only two occurred in Honduras, which were not found in El Salvador (H10, H11).

**Table 2:** Number of individuals used from each department in El Salvador or Honduras for mtDNA COI sequences

Country	No. of individuals	Department	Municipality/neighborhood
El Salvador	16	Santa Ana	Santa Ana
	10	San Vicente	San Vicente
	4	Cabañas	Sensuntepeque
	10	La Unión	La Unión, Santa Rosa de Lima
	5	Ahuachapán	Concepción de Ataco
	12^a^	Sonsonate	Sonsonate
	10^a^	San Salvador	San Salvador
	10^a^	Chalatenango	San Ignacio
	14^a^	Usulután	Jiquilisco
	16^a^	San Miguel	San Miguel
	20^a^	Morazán	San Francisco de Gotera
Honduras	10	Francisco Morazán	Tegucigalpa
	8	El Paraíso	Danli
	9	Choluteca	Choluteca
	2	Cortés	Cofradía
	8	Cortés	Los Angeles
	8	Cortés	Villanueva
Total	172	15	17

For El Salvador, 45 new sequences were produced, and 45 additional sequences were produced for Honduras. The 90 new sequences were combined with 82 previously sequenced (^a^see methods for GenBank numbers), for a total of 172 sequences analyzed

**Table 3:** Number of segregating sites (S), number of haplotypes (H), haplotype diversity (Hd), and nucleotide diversity (Pi) for each of the 17 regions included from El Salvador and Honduras

	Population (municipality)	S	H	Hd	Pi	Tajima’s D
El Salvador						
1	Ataco	9	2	0.600	0.012	1.777
2	Sensuntepeque	0	1	0.000	0.000	^–^
3	La Unión	10	6	0.889	0.011	1.781
4	Santa Ana	9	2	0.458	0.009	1.913
5	San Vicente	9	5	0.666	0.005	−1.320
6	Jiquilisco	9	3	0.538	0.009	1.601
7	San Francisco Gotera	15	7	0.773	0.013	1.455
8	San Ignacio	9	4	0.778	0.009	1.585
9	San Miguel	12	6	0.733	0.009	0.678
10	Sonsonate	9	2	0.167	0.003	−2.016^*^
11	San Salvador	15	5	0.867	0.015	1.223
		(El Salvador)	17	0.719	0.011	1.85 ns
Honduras						
12	Tegucigalpa	0	1	0.000	0.000	^−^
13	Villanueva	8	2	0.250	0.004	−1.701^*^
14	Danli	2	2	0.250	0.001	−1.310
15	Choluteca	9	2	0.222	0.004	−1.823^*^
16	Los Angeles	0	1	0.000	0.000	^−^
17	Cofradia	0	1	0.000	0.000	^–^
		(Honduras)	4	0.133	0.002	−2.00^*^
Overall values		19	19	0.637	0.010	1.108

*Statistical significance (*P* < 0.05). ^–^ Tajima’s test not calculated

In El Salvador, 10 haplotypes (H5–7, H9, H14–19) were found only in one population each, and 7 (H1–4, H8, H12–13) were shared among populations. The highest haplotype numbers and diversities were observed in three municipalities in the eastern portion of El Salvador, in San Miguel, Morazán, La Unión, and in the capital San Salvador (Table 3). Interestingly, lower haplotype diversity was observed in the Western portion of the country, in Santa Ana, Sonsonate and Ahuachapan, all having only the two most abundant haplotypes, H1 and H2. For Santa Ana, two third of samples were H2 (11/16), while Sonsonate was almost entirely H1 (11/12), and Ahuachapan was a mix of both H1 and H2 (Table 3, Fig. 4).

In Honduras, there were a total of four haplotypes; one haplotype (H1) was shared across all six populations, and three were only found in one population each. In Honduras, nearly all the samples (93%, 42/45 samples) were haplotype H1 (Fig. 4). This same haplotype was widespread as well in El Salvador in 9 of 11 municipalities and comprised 51% (52/127) of samples in El Salvador.

In El Salvador, a second haplotype H2 was also widespread in 10 of 11 municipalities and comprised 31.5% (40/127) of samples. Haplotype 2 was more frequent in individuals from Santa Ana and San Vicente and was also present at the rest of the localities of El Salvador, except inland in San Miguel (Fig. 4). Haplotypes 4, 8, and 13 were the next most widespread haplotypes and were shared by individuals from the SM, JI, LU, SV, SS, and SFG localities (Fig. 4). At the periphery of the network were haplotypes H3, H9, H10, H12, H13, H15, H18, and H19.

Assessment of population expansion based on the neutrality test found most values were not statistically significant. The overall Tajima’s D for El Salvador populations was 1.85, which was positive but nonsignificant. In El Salvador, only Sonsonate (ES) had a negative significant Tajima’s D (−2.016). In Honduras, Tajima’s D overall was −2.0 (Table 3). In addition, Tajima’s D values were negative and statistically significant in Villanueva (HON) (*D* = *−1.701*) and Choluteca (HON) (*D* = *−1.823*) (Table 3).

The Global AMOVA test was significant with 71% of genetic variation found within the 17 combined populations and 29% among populations (Table 4). AMOVA for El Salvador populations found a similar result of significant variation among populations, while one of the populations for Honduras found no significant variation among populations. The Fst values were highest and significant primarily between both Sensuntepeque (Cabanas-ES) and San Ignacio (ES) and other populations, as well as between most collections, compared to those from Honduras (Additional File, Supplemental Table 1).

**Table 4:** Analysis of molecular variation (AMOVA) among seventeen populations from El Salvador and Honduras

Source	df	Sum of squares	Variance components	Variation (%)	*P*
Among populations	16	275.658	0.69207	28.99075	0.0000
Within populations	155	525.493	1.69514	71.00925	
Total	171	801.151	2.38721		

## Discussion

*Aedes aegypti* were collected in El Salvador and Honduras, two adjoining countries in Central America. In El Salvador, a high haplotype number and diversity were found, while in contrast Honduras had few haplotypes. The haplotype H1 was most abundant and widespread in both countries. The most widespread haplotype (H1) in both countries may have been present the longest in both countries and perhaps is a remnant of eradication programs from the 1950s and 1960s [10]. Understanding the distribution of genotypes of *Ae. aegypti* in the region may contribute to management of this species.

Two predominant haplotypes, H1 and H2, were identified from the combined 17 populations. The current study included GenBank accessions obtained from samples in a previous study in El Salvador [15]; the study included a phylogenetic analysis which found that H1 and H2 belonged to two distinct *Ae. aegypti* mitochondrial lineages [15]. One lineage contains the widespread H1 haplotype, and the second lineage contains the H2 haplotype [2, 15]. Both lineages have been dispersed into many locations worldwide. The two most abundant haplotypes, H1 and H2 (and their associated lineages), could indicate separate invasions at different times. A number of other studies have similarly found two lineages of *Ae. aegypti* in the Western Hemisphere. In Panama, Eskildsen et al. (2018) recovered two deeply divergent mitochondrial clades, and similarly two genetic lineages were found in Bolivia [36]. Others studying South America similarly suggest the introduction of at least two *Ae. aegypti* lineages. In Peru, Venezuela, and in the Amazon, two *Ae. aegypti* mitochondrial lineages were detected [37–39]. These are all consistent with our results from El Salvador and Honduras.

In Honduras, nearly all individuals were haplotype 1 (H1). Haplotype 10 (H10), found in Northern Honduras in the department of Cortés, showed the greatest genetic distance from populations in El Salvador and southern Honduras. Geographically, this locality is characterized by its proximity to Puerto Cortés on the Honduran coast and Puerto Barrios on the Guatemalan Atlantic coast. There may be passive transport of eggs through ports into the department of Cortés Honduras, which would incorporate new alleles in this locality, causing an increase in its divergence from other populations. Results of the current study agree with the previous study in Honduras of *Ae. aegypti* [16], but the sampling region to date in Honduras is limited. Additional collections from other regions of the country, such as the north and eastern region, would provide more information about the genetic diversity of *Ae. aegypti*.

A high genetic diversity of *Ae. aegypti* might be expected in both countries, given the international trade between each country and exterior countries, which would provide the means for potential introduction of additional *Ae. aegypti* genotypes. The widespread distribution of the haplotype H1 through both countries suggests that the haplotype may have been present longer in the region than other haplotypes. One potential explanation for this could be that H1 is a remnant of eradication programs.

Populations with the highest genetic diversity indices were mostly in the east of El Salvador, in La Unión, San Francisco Gotera, San Miguel, and the capital San Salvador. La Unión (ES) has an old port and is characterized by the commercial flow with two neighboring countries, Honduras and Nicaragua, which could facilitate introductions of eggs via the Gulf of Fonseca. San Miguel and San Salvador (capital of ES) also have high commercial interconnectivity with the rest of the country. In addition, ecological factors may contribute to the observed genetic diversity. The private haplotypes observed in the network could have originated from factors such as local radiation, the adaptive processes associated with colonizing new habitats. The eastern region of the country including San Miguel and La Unión has high temperatures which facilitates rapid generation time for this mosquito species. Along with high temperatures, abundant mosquitoes and insecticide control, both anthropogenic and ecological factors could facilitate the production and movement of distinct haplotypes in this region [40, 41].

The Tajima’s D for El Salvador was positive but not significant (1.85), suggesting some genetic differentiation is occurring [42]. In addition, many populations in ES had unique haplotypes. Only Sonsonate (ES) had a significant negative Tajima’s D value. In Honduras, Tajima’s D was −2.0 overall, and values were negative and significant for Villanueva and Choluteca, indicating a recent bottleneck or selective sweep [42]. The AMOVA among all populations combined was significant, indicating gene flow among populations. However, the AMOVA for Honduras found most variation was within populations rather than between them.

This study has some limits and would benefit from additional related research. Mosquito samples could be obtained throughout additional regions of Honduras to better understand the genetic variability of *Ae. aegypti* through a wider region of the country. Samples from Honduras came mostly from an interior region of the country. Results from the current study suggest that sampling method may impact detection of genotypes. In El Salvador, larval samples were obtained for all populations, and a large number of haplotypes were found. In Honduras, collections originated from ovitraps and less genetic diversity were detected. Haplotype H1 was the most abundant haplotype found in Honduras, whereas El Salvador had an abundance of H1 and H2, along with numerous other haplotypes. A future study in Honduras might include larval collections and could examine if trap type influences the haplotype of *Ae. aegypti* collected.

## Conclusions

It is important to note that one genotype, H1, was widespread and abundant in both Honduras and El Salvador and present at nearly all collection sites. To further investigate whether H1 is a remnant of previous eradication programs, *Ae. aegypti* sequences from other countries in the Americas could be reexamined to determine whether this particular haplotype is more abundant and widespread in other regions which were under eradication. In El Salvador, H2 was the second most abundant type after H1. It is also possible that these abundant and widespread genotypes, H1 and H2, have persisted because of significant insecticide resistance [43, 44]. This could be investigated and may benefit management. Currently, *Wolbachia*-infected *Ae. aegypti* mosquitoes are being implemented in El Salvador and Honduras to reduce populations of *Ae. aegypti* [45]. The current study provides a foundation to understand the genetic variants of *Ae. aegypti* present in the region before these mosquito releases occurred. Understanding the mating frequency between the two mitochondrial lineages of *Ae. aegypti* (H1 and H2) may also be helpful and could contribute to the success of novel mosquito management strategies that rely on cross mating among populations. There remains a constant global challenge to control *Ae. aegypti* and reduce its populations to prevent vector borne disease.

### Supplementary Information

Additional File1 Table S1. Pairwise FST distance among populations. Populations from El Salvador: Ataco (AT), Sensuntepeque (SEN), Jiquilisco (JI), La Unión (LU), San Francisco Gotera (SFG), Santa Ana (SA), San Ignacio (SI), San Miguel (SM), Sonsonate (SON), San Salvador (SS), San Vicente (SV). Populations from Honduras: Tegucigalpa (TEG), Choluteca (CHO), Cofradia (COF), Villanueva (VIL), Danli (DAN), Los Angeles (LA). Collection locations of all populations are detailed in Table 1.

## Data Availability

The 90 new sequences generated during the current study are available in GenBank (El Salvador sample accession numbers PP732749-PP732793, Honduras sample numbers PP732794-PP732838). The 82 existing additional accessions (sequences) from GenBank included in the analysis were the following from El Salvador: Usulatán (MK028219-32), Morazán (MK028233-52), Chalatenango (MK028 253-62), San Miguel (MK028263-78), Sonsonate (MK028279-90), and San Salvador (MK028291-300)].
