# Supplementary material for: Genetic variability of Aedes aegypti (Diptera: Culicidae) in El Salvador and Honduras: presence of a widespread haplotype and implications for mosquito control
Source: Parasit Vectors. 2024 May 16;17:229. doi: 10.1186/s13071-024-06312-7 (PMC11100194; doi:10.1186/s13071-024-06312-7)
Supplement: Supplementary file 1 — Additional File1 Table S1. Pairwise FST distance among populations. Populations from El Salvador: Ataco (AT), Sensuntepeque (SEN), Jiquilisco (JI), La Unión (LU), San Francisco Gotera (SFG), Santa Ana (SA), San Ignacio (SI), San Miguel (SM), Sonsonate (SON), San Salvador (SS), San Vicente (SV). Populations from Honduras: Tegucigalpa (TEG), Choluteca (CHO), Cofradia (COF), Villanueva (VIL), Danli (DAN), Los Angeles (LA). Collection locations of all populations are detailed in Table 1. [file 13071_2024_6312_MOESM1_ESM.docx]

**Additional File, Supplemental Table 1.** Pairwise FSTs distance among populations. Populations from El Salvador: Ataco (AT), Sensuntepeque (SEN), Jiquilisco (JI), La Unión (LU), San Francisco Gotera (SFG), Santa Ana (SA), San Ignacio (SI), San Miguel (SM), Sonsonate (SON), San Salvador (SS), San Vicente (SV). Populations from Honduras: Tegucigalpa (TEG), Choluteca (CHO), Cofradia (COF), Villanueva (VIL), Danli (DAN), Los Angeles (LA). Collection locations of all populations are detailed in Table 1. *p<0.05, **p<0.01. ***p<0.001

**Table 5.** Analysis of molecular variation (AMOVA) among seventeen populations from El Salvador and Honduras.

| Source | df | Sum of squares | Variance components | Variation (%) | *P* |
| --- | --- | --- | --- | --- | --- |
| Among populations | 16 | 275.658 | 0. 69207 | 28.99075 | 0.0000 |
| Within populations | 155 | 525.493 | 1.69514 | 71.00925 |  |
| Total | 171 | 801.151 | 2. 38721 |  |  |
